# Does organizational justice facet matters in knowledge hiding?

**DOI:** 10.1016/j.heliyon.2023.e18372

**Published:** 2023-07-17

**Authors:** Hamid Mahmood, Asad Ur Rehman, Irfan Sabir, Abdul Rauf, Asyraf Afthanorhan, Ayesha Nawal

**Affiliations:** a TIMES Institute, Multan, Pakistan; b Management & Science University, Shah Alam, Malaysia; c University of Central Punjab, Lahore, Pakistan; d Faculty of Business and Management, Universiti Sultan Zainal Abidin, Kuala Nerus, Terengganu, Malaysia; e Operation Research & Management Sciences Research Group, Faculty of Business and Management, Universiti Sultan Zainal Abidin, Kuala Nerus, Terengganu, Malaysia; f Faculty of Business and Management, University Sultan Zainal Abidin, Kuala Nerus, Terengganu, Malaysia

**Keywords:** Organizational justice, Knowledge hiding, Professional commitment, Well-being, Job performance

## Abstract

To address the gap of extant literature and to assess employees' in-role and innovative performance, a model was developed and tested through organizational justice facets— procedural, distributive, and interactional justice with knowledge hiding facets, well-being facets and professional commitment. The purpose of the present research is to inspect the role of justice facets in shaping knowledge hiding behavior through optimistic role of well-being toward employee performance with the remedial role of professional commitment under the shadow of Psychological Ownership Knowledge Theory (POKT) and Social Exchange Theory (SET). For that persistence, present research acknowledged the practices and connotations of knowledge hiding because limited research is prevailed on the contrasting influence of knowledge hiding practice. Data were collected through random sampling via dual-wave survey questionnaire from 613 employees working in Kuala Terengganu, Malaysia. Structural Equation Modeling was carried out through AMOS (24.0) and SPSS (25.0). Findings reveal that the association with in-role and innovative performance with justice is positively associated through well-being, and the relationship between knowledge hiding and job performance was also positively associated. This study argued that knowledge sharing reshapes knowledge hiding behavior that plays a negative role in organizational performance. This study suggested the notable contribution in the direction of organizational context of developing realm settings by revealing the predecessor character of knowledge hiding and endorses the organizational justice to persuade top management for in-role and innovative performance enhancement.

## 1 Introduction

Nowadays, the use of excessive information, evolving recognition, transformation, intellectual capital, and knowledge have become a critical basis of competitive advantage [1,2]. Knowledge is recognized as a valuable and advanced intangible asset in world economies [3]. Organizations recently had enhanced their efforts to transfer knowledge, but there is remain of ineffective of knowledge management [4], and uncertainty about outcomes found against these efforts [5]. Initially, knowledge hiding attracted the devotion of anthologists and administrative sociologists in 1960 [6]. But since the last decade, researchers have focused on the behavior of knowledge hiding from an organizational perspective [7,8]. The knowledge hiding at organizational level has been found unfavorable for organizational functioning [9], to reduce growth potential and effectiveness [10], encumber elemental performance [11], challenges organization productivity and ingenuity by Ref. [12], and leads to hostile retaliation [13]. Some individuals are hesitant or unwilling to share knowledge with their associates [14,15] because they fear losing status or power via sharing knowledge with others [8]. Knowledge hiding is reported by many organizations [16], which is a deliberate endeavor to obscure or suppress knowledge by an individual that is demanded by another person [12].

According to Ref. [17], 46% of the total respondents from China believe that they hide knowledge from them even after requested [12]. revealed that 76% of the United States respondents hide knowledge to maintain their status [18]. stated that almost 500 companies bear a $31.5 billion loss due to knowledge hiding every year [19]. Knowledge sharing is an essential aspect of an organization's performance, and an individual's performance has keen interest from both organization and employees at large [20]. Organizations are keen to know how employee performance can improve, and employees are also keen to find factors that could improve their performance. Employees perform activities to reflect their preferences, capabilities, and perception regarding role holders [21]. Employees' well-being is in connotation between knowledge hiding and job performance [22]. Although, most individuals' tendency distinguishes them as generous and honest [23], and it could be problematic for individuals who exercise knowledge hiding and at the same time maintain their well-being. Employees' well-being decelerates and decreases optimistic knowledge hiding behavior and job performance [22].

An extensive amount of organizational knowledge is controlled at individual level. It is very important for policymakers and managers to make strategies to control knowledge hiding behavior of the employee, especially in knowledge intensive sectors. Past researches revealed antecedents of knowledge hiding factors [24] including situational factors [12], interpersonal factors [13], and personality traits [9]. However, organizational justice facets provide us more understanding of when and why knowledge hiding behavior occurs, which ultimately effect employee well-being and performance. Thus, the main motivation of this study is to explain organizational justice effect on knowledge hiding behavior that ultimately affect the employee performance.

Though, such contributions reassuring the precarious gaps can be filled to develop this research line in the context of job performance. Due to the growing connotation of such a problematic agenda of knowledge hiding, present research discourses some significant research calls [10]. To advance theory, practices and research on how managers mitigate the knowledge hiding effect, it is vital to know the source and nature of knowledge hiding based on organizational justice. Multiple aspects have been independently used to suggest the usage of psychological ownership theory [17,25] and social exchange theory [26]. Additionally, to best of our knowledge, no study considers both theories together to explain knowledge hiding. Thus, the aim of this study is to the address possibility by which psychological ownership theory and social exchange theory relate together to predict knowledge hiding. The possibility to use both theories together seems neglected so far. Lately a new study by Ref. [27] highlighted the lack of examining POK (Psychological Ownership Knowledge) with sharing knowledge. This paper is in response to the theory that knowledge hiding makes employee feel good because it assists to punish the unfair operant in social settings [28].

Present research emphasizes the justice-based relationship between employees and management, and professional commitment paving the way for triggering justice and demoralizing knowledge hiding behavior. Relied on above identified gap, present research aims to 1) identify the negative impact of procedural, distributive, and interactional justice on knowledge hiding, 2) examine the negative impact of knowledge hiding on employees' well-being, 3) explore the positive impact of employees' well-being on innovative and in-role performance, 4) analyze the mediating effect of knowledge hiding and employees' well-being on the relationship between organizational justice facets and employees’ well-being, and knowledge hiding and employees in-role and innovative performance respectively, and 5) find out the negative moderating effect of professional commitment on the relationship between procedural, distributive, and interactional justice and knowledge hiding. Based on above argument, consequently, the relationship is weaker when the professional commitment is higher rather than low among the auto mobile parts manufacturing organizations. So, employees who have knowledge are unable to decide which type of knowledge can shared or concealed. Present research offers value-based model to holistically understand the phenomenon of KH that cure with justice and professional commitment for triggering employee performance. We advocate that employee might take self-enhancing actions at that time, which harm organizational performance and growth [24] that recommends investigating explicit knowledge hiding despite the universal behavior of knowledge hiding.

## 2 Literature review

### 2.1 Theory and hypothesis development

#### 2.1.1 Psychological ownership knowledge theory and social exchange theory

Psychological ownership is an attitude to which individuals feel that the ownership of target or some part of target is theirs only [29,30]. Ownership can theorize both as psychological state or an objective perspective [31,32]. It decrees extra role behavior by countering behavior amid incumbent employees [33]. In knowledge context, POKT amid incumbent workers, knowledge ownership feelings and its possession [34], have tendency to predict knowledge hiding or sharing [8]. Knowledge hiding happens through either, expected loose of control or overestimating of the knowledge [10]. Social exchange theory (SET) explains the dynamics in transaction and relationship among two or more participants [35]. The intervention in the process of exchanging resources among organization and employees can affect their behavior and attitude [36]. Hence, positive dealing of the organization in form of justice would alter with positive behavior of the employee, like knowledge sharing [37] or vice versa. While organization or colleagues treat them unfairly (i.e., Knowledge hiding) they suppress discretionary positive attitude [38].

### 2.2 Organizational justice facets and knowledge hiding

Fairness is considered the most imperative factor in many spheres of individual lives; therefore, it has received much attention from social science scholars [39] and practitioners for many years [40]. Employees' fairness perception with cognitive, behavioral, and emotional reactions refers to organizational justice [41]. According to Ref. [42], a most leveraged method to understand the concept introduced that organizational justice facets with an employee are agitated about the fortitude of how employees are treated impartial manners that affect the work-related variables fortitude. Organizational justice is considered a significant predictor of knowledge hiding behavior of the employees [43]. Furthermore, employees' knowledge hiding behavior is minimized in a harmonious context. And, organizational regulation plays a vital role in decreasing the knowledge hiding practices of employees [43]. Organizational justice facets— procedural, distributive, and interactional justice is an essential factor contributing to organizational regulation, which may decline employee's knowledge hiding behavior [43].

#### 2.2.1 Procedural justice

Procedural justice refers to the equitable decision and fair allocation in terms of procedure, dispute settlement, and process [39,44]. It entails employees' perception of mechanism, motives, and methods used to control the outcomes or absolute impartiality of processes involved in decision building [16]. Generally, procedural justice is considered a pressing issue in any organization. Positive perception of procedures and processes to determine the performance is connected with a higher degree of trust in its managers and organizations [45]. Conversely, extant studies established negative consequences of dissension of procedural justice [46]. Indeed, procedural justice concerns may risk producing dissatisfaction with decisions of organizational outcomes, non-compliance with procedures and rules, negative organizational attitude, and low performance [47]. The fairly treated feelings by the organization enhance the confidence and trust of the employees on procedures and decisional process, which encourages a positive attitude towards the workplace [48]. Fair procedure illustrates organizational dignity, respect, and rights of the employees. Procedural justice exaggerates knowledge-sharing behavior [36] because discretionary behavior, such as sharing knowledge enhanced when employees are treated sincerely by the organization [49]. Procedural justice in an organization leads to both employees’ and organizational justice, like avoiding knowledge hiding [36].

Hypothesis 1a Procedural justice has a negative impact on Knowledge Hiding.

#### 2.2.2 Distributive justice

Distributive justice reflects fairness in distribution like promotions, benefits, resources, and salary [39]. Employees develop perceptions of fairness in distribution by comparing themselves with others [50]. Distributive justice in an organization provides safety to employees over equitable allocation of resources, which satisfy and increase obligation sense [51], to counter negative attitude of employees [52]. According to Ref. [53], unfair distribution may have dreadful consequences, including social problems, disputes, and disrespect among employees. Furthermore, partial distribution of possessions and opportunities diminish the spirit of employees. According to Ref. [54], this advocates a crucial prerequisite to maintain fairness when procedures are violated, and rules are absent, similarly when workers practice knowledge hiding. Depending on related outcomes and cooperation, distributive justice discourages knowledge hiding behavior among employees [36].

Hypothesis 1b Distributive justice has a negative impact on Knowledge Hiding.

#### 2.2.3 Interactional justice

[55] introduced the interactional justice concept, which advanced justice literature that embraces interpersonal interaction qualities among employees. Interactional justice focuses on delivering information to the employees regarding different procedures and practices to define why specific procedures are followed [39]. It shapes reactions towards their supervisor, peers, and immediate work environment [56]. Process implementation methods and employees’ interaction emphasize the process and attitude of an organizational employee [57]. Interactional justice occurs when superiors treat employees with interpersonal dignity, discuss implications, participate in simple transactions, explain and justify all activities [44]. Social interaction is a lifeline for the quality of workplace, which may stagger other organizational domains.

Consequently, employees’ perception for interpersonal treatment and interaction shapes numerous employees and organizational outcomes. According to Ref. [36], an employee may hide knowledge from peers, superiors, or others with whom they interact. Additionally, this justice is an essential tool that was positively subsidizing to advance administrative discipline by declining knowledge hiding behavior [43].

Hypothesis 1c Interactional justice has a negative impact on knowledge hiding.

### 2.3 Knowledge hiding and well-being

Knowledge hiding is a cautious effort by the employee to withhold specific knowledge requested by and another employee. Playing dumb, evasive hiding and rationalized hiding are the facets of knowledge hiding stated by Ref. [12]. Knowledge hiding behavior differs from other dyadic behavior, such as deception, workplace aggression, knowledge hoarding, territoriality, social undermining, workplace incivility, and knowledge sharing [18]. Consequently, knowledge hiding has diverse implications and antecedents for organizations and individuals. Although sharing information appears necessary and expected in some organizations, but in many organizations, knowledge hiding happens for prosocial reasons and as well as self-interest; therefore, knowledge hiding behavior becomes fewer offensive norms [22]. Employees strategically hide the knowledge to exploit their value or act with organizational harmony and norms [10].

Additionally [58], argued that employees sometimes hide their knowledge without specific pressure which harms their well-being. According to Ref. [23], numerous employees engaged in the knowledge hiding behavior might encounter psychosomatic distress and enervation. Based on the above arguments, it's rational to suppose that knowledge hiding negatively influences employees' well-being.

Hypothesis 2 Knowledge hiding has a negative impact on employees' well-being.

### 2.4 Well-being and job performance facets

[59] argued that well-being is a complex variable defines as *"a function of the actual conditions of [one's] life and what an individual makes of those conditions"* (pp. 1–6). Many scholars studied well-being with different aspects and components such as physical, economic, emotional, and social well-being. Two concerning prospective of well-being— subjective hedonic and physical eudemonic are to be found in past literature [60]. Eudemonic well-being reflects perceived development, a sense of purpose, self-discovery, maximum ability of an individual, investment of momentous effort, enjoyment of events as an expression of one's life, intense involvement in activities, and meaningful life [61]. The focus of our study is eudemonic well-being; nevertheless, it addresses employee intention, direction, and goals that contribute to making their life meaningful [62], and it also advocates efficiency and stability in organizational functioning. Well-being found a positive effect on job performance [60]. Job performance plays a vital role in the success of the organization and employees as well. Job performance is defined as a set of behavior demonstrate by an employee to contribute indirectly or directly to achieve organizational commitment [63]. Additionally, high job performance provides positive benefits to the employee, such as better career, high income, and social reputation [64]. Job performance is a complex and broad construct with two essential facets— innovative and in-role performance [22].

#### 2.4.1 Innovative performance

Innovation is defined as the adoption or generation of novel and valuable ideas that are effectively introduced in an organization [65] and, has equal importance in the success of employees and organization [66]. An employee can bring creative and new ideas to solve business problems [67]. However, the capacity to innovate is an essential factor affecting organizational performance and strategic achievement and goals [68]. Innovative performance has a decisive contribution to organizations' competitive advantages [69]. stated that organizational factors could enhance innovative performance, and according to Ref. [70], employee strategic orientation can also enhance the innovative performance.

Furthermore, employees' innovative capabilities positively enhance the organizational performance. Organizations need employees to be committed, satisfied, and optimize their efforts with organizational commitment, and enhance innovation at the workplace [71]. Although, for better performance and sustainability, organizations need to assess the importance of the innovation from employees [72]. Consequently, innovative performance is a predicted construct of well-being that enhances performance [73]. Based on the above discussion, an employee's well-being is expected to contribute toward attitude, intrinsic motivation, thinking style, and self-efficacy, that ultimately enhancing innovative performance.

Hypothesis 3a Employees' well-being has a positive impact on innovative performance.

#### 2.4.2 In-role performance

In-role job performance is defined as activities related to employees' tasks or formal role requirements specified in job portrayal [74]. The job performance (in-role) assures the predictability of work performance, and organizations rudimentary tasks are regulated and synchronized to achieve organizational objectives [75]. It considers an essential factor in overall performance since it focuses on the requirement and expectations of individual roles [76]. Based on extant studies, when moral actions from leaders for the well-being of employees are a critical component of organizational goals and vision, employees tend to follow these actions, and their effort translates into in-role performance [77,78]. In-role performance is considered a primary indicator of employees’ motivation and organizational performance, which is influenced by well-being [22].

Hypothesis 3b Employees' well-being has a positive impact on in-role performance.

### 2.5 Knowledge hiding and well-being as mediators

According to Ref. [79], employees can practice the knowledge hiding facets when peers requesting particular knowledge and such practice bitterly affect the employees' well-being at workplace. To do so, the perpetrator (knowledge hiding practitioners) disseminates not as much of knowledge that as per required or inappropriate info [12,23]. Consequently, it speculates that optimistic implementation of organizational justice at workplace minimize the behavior of employees' knowledge hiding that ultimately be the cause of employee's overall well-being. Empirical evidence prevailed that relationship among exploitative leadership and task performance justified through the mediating role of knowledge hiding with the support of SET (social exchange theory) [22]. Furthermore, the mediated link of knowledge hiding put an optimistic role between employees scoring greatly on adverse reciprocity credence [80]. On the other hand, the facets of well-being like psychological, workplace, and life [81] refers to the approach of eudaimonic regarding psychological well-being and employees' hedonic views regarding workplace and life well-being [82]. While considering the well-beings’ low levels instigated by interactive conflict at workplace may activate the behavior of employees' knowledge hiding that minimizes the employees' well-being by depleting the individuals' mastery sense [83], so, employees practice the behavior of knowledge hiding to reinforce typically vanished resources. An extant study of [79] revealed that well-being as a mediator played a remedial role among knowledge hiding and interactive conflicts at workplace for the sake of performance enhancement.

Hypothesis 4a Knowledge hiding mediates the relationship between organizational justice facets— procedural, distributive, and interactional justice and employees' well-being.

Hypothesis 4b Employees' well-being mediates the relationship between knowledge hiding and employees' in-role and innovative performance.

### 2.6 Professional commitment as moderator

According to Ref. [84], the extant studies exclusively focus on the organizational commitment that refers to the commitment toward the organization but scarcely practice the professional commitment that denoted the commitment toward the profession. Therefore, we considered the broader hitherto correlated ideas of commitment toward profession [85] because empirical evidence shows that a sound association exists among sentimental, inflexible, normative, and endurance commitment toward both the organization and the profession, along with mutual backgrounds and comparable consequences. Hence, we measured professional commitment instead of organizational commitment [86] that contributes to the current literature and improve assurance among the associations of both extensive constructs. An employee's performance is improved due to his/her particular behavior and societal environment in specific circumstances. So, professional commitment is retained due to its inclusive benefits for employees in different situations. Based on social cognitive theory, we employed professional commitment as a moderator for the theoretical foundation. Social cognitive theory is extensively pragmatic to elucidate individuals' behaviors toward various research fields like in management [87], in marketing by Ref. [88], in mass communication by Ref. [89], and in education by Ref. [90]. In the present research, professional commitment as a moderator plays a vital role in the relationship of organizational justice facets and employee's knowledge hiding [43].

**Fig. 1:**
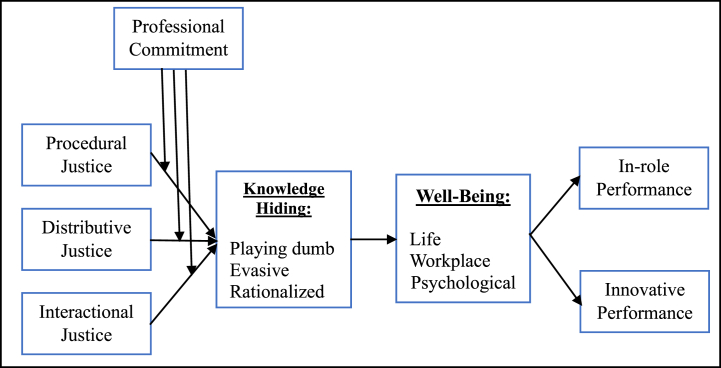
The research model. Source: Authors own conception.

The possibility exists that professional commitment fortifies the adverse correlation between organizational justice facets and knowledge hiding [91] because employees are trustworthy, enthusiastic, and have strong credence in pro-objectives and morals with high-level professional commitment [92]. According to Ref. [93], a great effort was shown among individuals through healthy, compassionate, and vicarious responsibilities. Based on the above arguments, individuals who were more committed to their profession might be heavily persuaded to share knowledge and be interested in assisting their coworkers. Therefore, professionally committed individuals desire to build their careers via ethics and allegiance [94]. So, the relationship between organizational justice facets and knowledge hiding might be moderated via professional commitment (See Fig. 1).

Hypothesis 5a Professional commitment negatively moderates the procedural justice effects on knowledge hiding, so the relationship is weaker when professional commitment is high rather than low.

Hypothesis 5b Professional commitment negatively moderates the distributive justice effects on knowledge hiding, so the relationship is weaker when professional commitment is high rather than low.

Hypothesis 5c Professional commitment negatively moderates the interactional justice effects on knowledge hiding, so the relationship is weaker when professional commitment is high rather than low.

## 3 Research methodology

### 3.1 Sample size

Data were gathered through random sampling from the employees of manufacturing industries in Kuala Terengganu, Malaysia. The focal persons of auto mobile parts manufacturing industries provided a list of 850 employees who currently work at that time. After the telephonic conversation, out of 850 employees, only 763 employees consented to participate in the survey and asked their email addresses for sending the questionnaire. Organizational justice with employee knowledge hiding behavior varies the perception of job performance across industries and professions. Data were collected from the heterogeneous sample for generalizability and to determine the maximum variation among knowledge hiding and job performance. A heterogeneous based sample assisted in capturing the maximum variation among the study's key constructs and heightened the generalizability of the findings [95,96]. Data were collected through random sampling at two-months interval to lessen the correlation bias, and common method variance was diagnosed using Herman's single factor [97]. The present study's constructs were constrained into one factor that explained 37.94% of the total variance that below the recommended value of 50% [98]. According to Ref. [17], short intervals affect respondents' ratings gained from the questionnaire's first wave compared to the second wave, which prejudice the critical association between knowledge hiding and job performance [99]. Contrarily, long intervals paved the way for risk to dispel the causal effect if the researcher takes much time to measure [100]. Based on the extant studies, a two-month lag was used to conduct the survey that seems to be a suitable length of lag time to meet the criteria.

### 3.2 Data collection procedures

Initially, with anonymity assurance, the questionnaire was sent to each respondent along with a cover letter. Participants were asked to respond to organizational justice facets, knowledge hiding, professional commitment, well-being, and demographic variables in the time one survey. Out of 763 distributed questionnaires, 613 questionnaires were received with an 80% response rate. The researchers voluntarily take assistance from four colleagues that assist in data collection process. Under the consideration of ethics, researchers assured the respondents that all the information keep confidential and use only for academic purpose. With the interval of two months at time two survey (second wave), 613 same participants were asked to respond on job performance (in-role and innovative performance). The employee's IDs were used as an identification code to link the first and second waves of the questionnaire. Consequently, 100% response was gained from the respondents of the two-wave survey. Out of 613 respondents, 63% were male, 61.5% fell between 25 and 31 years, 45.5% were graduates, 49.1% were workers, 41.8% had experienced between 5 and 9 years, and 73.7% were Malaysian nationals, and 77.8% were single.

### 3.3 Measures

In the present study constructs were measured at 10-point interval rating scale that ranges from (1) "strongly agree" to (10) "strongly disagree". According to Ref. [101], larger-scale like 10-point interval rating provide benefits; 1) offers more variance as compared to smaller scales; 2) offers a higher degree of measurement accuracy, and 3) provide an opportunity to detect changes and more power to explain a specific point of view. So, we choose 10-point interval rating as the most knowledgeable and satisfied service today [102]. The factor loading, AVE, CR, and instrument items are presented in Table 2 via using SPSS. Questionnaires were designed into two parts, the first part contained 55 close-ended questions, and the second part included the demographic profile of respondents. Organizational justice is measured as a higher order construct based on three dimensions with fourteen loaded items. Procedural justice was measured with five items adapted from Refs. [103,104]. Second, distributive justice was measured with four items adapted from Ref. [105]. Third, interactional justice was measured with five items from Ref. [106].

Knowledge hiding was measured based on three dimensions (playing dumb, evasive hiding and rationalized hiding) with twelve loaded items adapted from Ref. [12]. Well-being is also a higher order construct measured on three dimensions, i.e., life well-being, workplace well-being, and psychological well-being, with fifteen loaded items adapted from Ref. [81]. The professional commitment was measured based on six items adapted from Ref. [107]. At the same time, in-role performance was measured with six items adapted from Ref. [108]. Lastly, five items were adapted to measure the innovative performance [109]. The demographic profile of respondents was measured with age, gender, education, designation, experience, country and marital status presented in Table 1.

**Table 1:** Profile of respondent (n = 613).

Variables	Frequency	Percentage
Gender	389 224	63% 37%
Male		
Female		
Age	107 377 89 40	17.5% 61.5% 14.5% 6.5%
18–24		
25–31		
32–38		
39 & above		
Education	177 279 138 19	28.9% 45.5% 22.5% 3.1%
Diploma		
Graduation		
Master		
Others		
Designation	47 88 177 301	7.7% 14.3% 28.9% 49.1%
Senior Manager		
Manager		
Supervisor		
Worker		
Experience	89 156 256 112	14.6% 25.4% 41.8% 18.2%
Less than 1 Year		
1–4 Years		
5–9 Years		
10 Years & Above		
Country	452 161	73.7% 26.3%
Malaysia		
International		
Marital Status	477 136	77.8% 22.2%
Single		
Married

**Table 2:** Confirmatory factor analysis (n = 613).

Construct	Dimension	Indicators	SFL	AVE	CR
Procedural Justice (IV) α = 0.892, √AVE = 0.81		Are you able to convey your ideas during procedures?	0.95	0.661	0.905
		Are you influenced over (consequences) that arrived during procedures?	0.84		
		Are the procedures consistently applied?	0.61		
		Are all the procedures free from bias?	0.89		
		Is all information being accurate in these procedures?	0.73		
Distributive Justice (IV) α = 0.923, √AVE = 0.72		Does a (consequence) reflect your efforts at work?	0.75	0.663	0.887
		Are the (consequences) appropriate rendering your completed work?	0.82		
		Are (consequences) reflecting your contribution toward organization?	0.89		
		Are (consequences) being justified your performance?	0.79		
Interactive Justice (IV) α = 0.889, √AVE = 0.73		Are you treated in a polite manner?	0.78	0.539	0.853
		Are you treated with dignity?	0.73		
		Are you renounced from improper comments?	0.66		
		Are you thoroughly explained the procedures?	0.68		
		Can you reasonably explain the procedures?	0.81		
Knowledge Hiding (MED) α = 0.943, √AVE = 0.84	Playing Dumb	Admit to assist but never categorically intended.	0.85	0.712	0.881
		Consent to help but always give unwanted information.	0.86		
		Give irrelevant information instead of desired one.	0.82		
	Evasive Hiding	Acting that you have no information.	0.89	0.686	0.867
		Even you know but pretend that you did not know.	0.82		
		Said that what you are talking about even you have complete idea about it.	0.77		
	Rationalized Hiding	Pretend that you would like to tell something but were not supposed to.	0.83	0.723	0.887
		Pretend that you have confidential information and only gave to specific people.	0.84		
		Simply state that you wouldn't answer the question.	0.88		
Well- being (MED) α = 0.919, √AVE = 0.81	Life well-being	I feel that my life is satisfied.	0.76	0.661	0.907
		I feel that my life is close to my dreams.	0.82		
		I feel happiness in most of the times.	0.77		
		My life is in a good situation.	0.87		
		In afterlife, I hardly change my current life.	0.84		
	Workplace well-being	Regarding work responsibilities, I am satisfied.	0.82	0.667	0.909
		I generally feel that my present job is fairly satisfied.	0.92		
		I feel that my work is really enjoyable.	0.85		
		For me, work is an important practice.	0.72		
		In current job, I am satisfied with my achievements.	0.76		
	Psychological well-being	I am growing as an individual.	0.95	0.661	0.905
		I was soundly handling the daily affairs.	0.84		
		I usually feel self-confident and decent about myself.	0.61		
		There is a perception about me that I give my time to others.	0.89		
		For better understanding, I deeply conversation with my friends and family.	0.73		
Professional Commitment (MOD) α = 0.932, √AVE = 0.80		I have a great deal of exertion for professional development that beyond the expectation.	0.75	0.632	0.911
		I have a personality that strongly identifies my profession.	0.82		
		I generally accept any kind of job that related to my profession.	0.89		
		I have strong relations with my professional peers.	0.79		
		The profession which I belong feels me proud.	0.78		
		Regarding job performance, my profession actually inspired me.	0.73		
In Role Performance (DV) α = 0.823, √AVE = 0.79		I completed my assigned duties effectively.	0.66	0.626	0.908
		I fulfilled my job description responsibilities.	0.68		
		I always meet the formal job requirements.	0.81		
		I usually ignore my essential duties.	0.85		
		I feel heavy work load to help others.	0.86		
		I feel pleasure to solve the problems of coworker.	0.86		
Innovative Performance (DV) α = 0.913, √AVE = 0.78		It condenses the risk of innovation.	0.63	0.622	0.891
		It decreases the development cost of new product.	0.89		
		It diminishes the market time.	0.82		
		It instigates the newly developed services or products.	0.76		
		There is a greater acceptability of our products.	0.82		

Note: √AVE = discriminant validity, AVE = average variance extracted, SFL = standardized factor loadings, CR = composite reliability, α = Cronbach's alpha.

### 3.4 Statistical software for the research

According to Ref. [110], a questionnaire was developed to follow the method of back-translation, so the researcher translated the complete questionnaire from English into Malay with the help of a language expert and then re-translated them into English version with the help of a native speaker. The current research followed [111] procedure for measurement validation. The measurement model is valid when the standardized loadings, fitness indexes, reliability and validities assessments meet the acceptable values as recommended by previous scholars [100,[112], [113], [114]]. The poor standardized loadings were excluded from further analysis. The model is tested using the confirmatory factor analysis (CFA) and the results of fitness indexes are shown as follows: (χ2/df = 2.799, GFI 0.929, RMSEA 0.047). The chi-square test shows that both alternative models did not show the model fitness compared with the proposed model. Therefore, based on the above argument, we consider the hypothesized model to test the hypothesis. Convergence validity was tested using Average Variance Extracted (AVE) and the results for all construct meet the acceptable limits of 0.50. In additions to that, the reliability results also meet which is above 0.70 suggesting that all constructs involved in the present study are reliable. Then, the discriminant analysis is tested using Fornell- Larcker criterion. It was suggested that the model is distinct and free from any redundancy problems [115,116] (see Table 3).

**Table 3:** Discriminant validity.

Construct	Mean	SD	VIF	PJ	DJ	IJ	KH	WB	PC	IRP	IP
**PJ**	8.8109	.84202	1.236	**0.81**							
**DJ**	9.0824	.91308	1.576	0.30	**0.72**						
**IJ**	8.8783	.94610	1.113	0.59	0.71	**0.73**					
**KH**	8.8933	.94195	1.421	−0.28	−0.39	−0.42	**0.84**				
**WB**	9.2341	.88953	1.877	0.54	0.33	0.22	−0.23	**0.81**			
**PC**	9.0543	.89968	1.465	0.36	0.47	0.33	0.42	0.37	**0.80**		
**IRP**	8.9007	.92671	1.223	0.21	0.56	0.32	−0.47	0.51	0.37	**0.79**	
**IP**	8.9438	.91302	1.893	0.39	0.55	0.47	−0.31	0.49	0.44	0.17	**0.78**

Note 1: I.J. = Interactional justice, P.J. = Procedural justice, D.J. = Distributive justice, K.H. = Knowledge hiding, W.B. = Well-being, P.C. = Professional commitment, I.R.P. = In-role performance, I.P. = Innovative performance.

Note 2: The bold numbers in diagonal rows are square root of AVE.

## 4 Data analyses and results

### 4.1 Respondents’ profile

The profile of the respondents is summarized in Table 1. The higher number of respondents (63%) was male, 61.5% were fallen in the age between 25 and 31 years, and 77.8% were single. Approximately half (45.5%) had a graduation degree, 28.9% were diploma holders, and 22.5% had a master's degree. Nearly half (49.1%) had worked as a worker, 28.9% belonged to the supervisor's designation, 14.3% were managers, and only 7.7% were senior managers. Slightly less than half (41.8%) (n = 256) participants having experience of 5–9 years, 25.4% (n = 156) having 1–4 years' experience, and less than a quarter (18.2%) participants having experience of 10 years and above. Regarding the geographical point of view, we divided the participants into two groups, the maximum number of participants were from Malaysia (73.7%), and the remaining participants were internationals (22.2%) (n = 136) (Table 1).

### 4.2 Measurement model assessment

Latent constructs' reliability and validity were tested using two-step approaches [111]. Results of present study reveals that model fit indices are within the acceptable range that assured the measurement model confidence [RMSEA = 0.047, χ2/df = 2.799 [98]]; [AGFI = 0.856 [117]]; [NFI = 0.976, RFI = 0.962, GFI = 0.929, SRMR = 0.038, CFI = 0.981, TLI = 0.978 [98]]. CFA was used in the present study to examine the relationship among constructs, and the model was tested via structural equation modeling [118]. The threshold value of factor loading (0.60) was also achieved because all items are in-between 0.61 to 0.95, shown in Table 2. According to Ref. [119], the recommended value for composite reliability is 0.70. The present study's results reveal that all scores ranged between 0.823 and 0.943 as shown in Table 2. According to Ref. [115], the AVE must be higher than the cut-off value of (0.50), and the results of this study meet the threshold value of AVE. The measurement items' internal consistency was measured through Cronbach's alpha (α), and the present study results achieved the threshold value of 0.70. This study used a two-step approach to examine the discriminant validity below the threshold value of 0.85 [120].

### 4.3 Common method bias

According to Ref. [97] for the measurement of common method variance of each construct's, the Herman single factor was used. The variance counterfeit was assessed between the constructs because the common method was used for data collection. All the construct items' exploratory factor analysis revealed that the key three factors acquisitively rational for 63.88% of the variance between constructs, with major factor rating for 39.47% and the subsequent factor explanation 24.41% of the total variance. Hence, the variance of the first factor below the threshold level which is 50%. So, this data is pure from common method biasness.

### 4.4 Structural model

Present study hypotheses were tested using structural equation modeling through SPSS AMOS (24.0). Categorically, a bootstrapping technique was performed to identify the effects of organizational justice on knowledge hiding and the first mediator on second mediator well-being and both mediators in the association among organizational justice facets and job performance. Also, inspect the impact of moderator PC on the association of organizational justice facets and the knowledge hiding. Hypothesis tests' results are presented in Table 4. Results revealed that indices of goodness fit: (χ2 (178) = 498.289, χ2/df = 2.799, SRMR = 0.038, RMSEA = 0.049, GFI = 0.927, CFI = 0.986, AGFI = 0.843, NFI = 0.972, RFI = 0.966, TLI = 0.982). Based on the large sample size, the goodness of fit indices and value of ChiSq/df indicated an acceptable fit with the model. According to Ref. [119], the structural model results indicated that the association among constructs is capably considered. The present study model perfectly captures the 69% of in-role and innovative performance through exogenous constructs (organizational justice facets, knowledge hiding, well-being and professional commitment).

**Table 4:** Results of hypothesis.

Path	Standardized estimation	*t*-Statistics	*p-*value	Relationship
Procedural justice → Knowledge hiding	−.469	−8.242	<0.001	Supported
Distributive justice → Knowledge hiding	−.482	−7.180	<0.001	Supported
Interactional justice → Knowledge hiding	−.376	−6.954	<0.001	Supported
Knowledge hiding → Well-being	−.388	4.162	<0.001	Supported
Well-being → In-role Performance	.567	5.412	<0.001	Supported
Well-being → Innovative Performance	.375	2.559	<0.001	Supported
Procedural justice → Knowledge hiding → Well-being	−.388	−4.162	<0.001	Supported
Distributive justice → Knowledge hiding → Well-being	−.375	−2.559	<0.001	Supported
Interactional justice → Knowledge hiding → Well-being	−.467	−8.426	<0.001	Supported
Knowledge hiding → Well-being → In-role Performance	−.369	−2.551	<0.001	Supported
Knowledge hiding → Well-being →Innovative Performance	−.276	−2.339	<0.001	Supported
	Structural Model		Cut-off Value	
Model fit statistics	Chi-square = 498.289			
	df. = 178			
	p-value = 0.000			
Absolute fit index	Normed ChiSq = 2.799		1.0–3.0	
	RMSEA = 0.049		<0.08: good fit	
	GFI = 0.927		>0.90	
	AGFI = 0.843		>0.80	
Incremental fit index	NFI = 0.972		>0.90 >0.90 >0.90 >0.90	
	IFI = 0.978			
	CFI = 0.986			
	TLI = 0.982			
	RFI = 0.966			
Parsimonious fit index	PCFI = 0.745		>0.50	
	PNFI = 0.779		>0.50	
	PGFI = 0.655		>0.50	

### 4.5 Hypothesis results

To consider the issue of multicollinearity, we performed the test of variance inflation factor and found in-between the range of 1.113–1.893 that fall under the threshold value of 3.0 (Table 3). Based on present study findings, procedural justice negatively affects knowledge hiding (coefficient = −0.469, t = −8.242), supporting the H1a. Results indicate that being the procedural justice in the organization discourage knowledge hiding behavior among employees. H1b was supported because distributive justice negatively affects knowledge hiding (coefficient = −0.482, t = −7.180). The findings of H1b indicated that organizational distributive justice disappoints the knowledge hiding behavior between employees. Interactional justice negatively affects knowledge hiding (coefficient = −0.376, t = −6.954), so H1c was supported. H1c findings highlighted that interpersonal or interactional justice also discourages the behavior of knowledge hiding among employees. Present study findings were also consistent with the findings of [121] because organizational justice negatively affect the behavior of knowledge hiding. H2 was supported because knowledge hiding also negatively affects well-being (coefficient = −0.388, t = 4.162). Findings of present study was also consistent with the findings of [22]. H2 findings revealed that knowledge hiding plays a negative role in employee's well-being. Well-being positively affects the employee's in-role performance (coefficient = 0.567, t = 5.412), so H3a was supported. The findings of H3a pointed out that an employee's well-being plays a vital role in the employee's in-role performance. H3b was also supported because well-being positively affects the employee's innovative performance (coefficient = 0.375, t = 2.559). Present study findings were also consistent with the findings of [122] because employees' well-being positively affect the facets of employee performance. H3b findings revealed that well-being plays a motivational role to enhance the employee's innovative performance. Knowledge hiding mediates the relationship between organizational justice facets i.e., procedural justice and employee's well-being (coefficient = −0.388, t = −4.162), distributive justice and employee's well-being (coefficient = −0.375, t = −2.559), interactional justice and employee's well-being (coefficient = −0.467, t = −8.426) so, H4a was supported. The findings of H4a pointed out that an employee's knowledge hiding behavior plays a vital role in the relationship among organizational justice facets and employee's well-being. H4b was also supported because well-being mediates the relationship between employee's knowledge hiding behavior and employees' performance facets i.e., in-role performance (coefficient = −0.369, t = −2.551), and innovative performance (coefficient = −0.276, t = −2.339). Present study findings were also consistent with the findings of [123,124] because well-being and knowledge hiding played a mediational role and robustly influence the relationships. H4b findings revealed that well-being plays a motivational mediational role among knowledge hiding and the employee's in-role and innovative performance (Table 4).

**Fig. 2:**
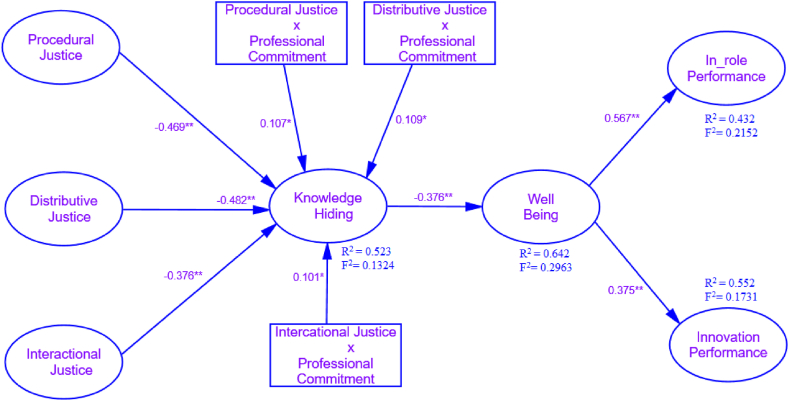
Standardized structural equation parameter estimates. Source: Based on author's own conception after calculation on AMOS software.

The total variance in the dependent variable via changes in the independent variable is signposted through the R^2^ value, and results highlighted a 52.3% change in knowledge hiding, 64.2% in well-being, 43.2% in in-role performance, and 55.2% in innovative performance. According to Ref. [119], the recommended value of R^2^ is 60% that indicates the total variance of all endogenous variables. According to Ref. [125], the research's model substantial effect was observed via checking effect size (ƒ^2^) and recommended rule of thumb for small, medium and large threshold is 0.02, 0.15 and 0.35, respectively that denoted with an existed object between populations. Present study model revealed that knowledge hiding (ƒ^2^ = 0.1324) has a small effect size, well-being (ƒ^2^ = 0.2963), in-role performance (ƒ^2^ = 0.2152), and innovative performance (ƒ^2^ = 0.1731) has a medium effect size (Fig. 2).

### 4.6 Moderation and the simple main effect

The metric variables moderation was tested through interaction effects. Even interaction effect was significantly attained; we further explored the interaction nature via testing simple effects, so we followed [126].

Initially, we test the procedural justice and professional commitment direct effect on knowledge hiding. Results revealed that procedural justice direct effect on knowledge hiding was statistically substantial (F = 11.556), and the interaction effect between procedural justice and professional commitment (procedural justice x professional commitment) on knowledge hiding are also statistically substantial (β = 0.107, t = 2.456). Via using dummy variables, the moderator variable dataset was divided into two groups; low and high. So, we test the procedural justice on knowledge hiding at both low and high levels of professional commitment. As expected, procedural justice negatively affects knowledge hiding while having a high-level professional commitment (β = −0.581, t = −5.721). Procedural justice is positively related to knowledge hiding while having low-level professional commitment (β = 0.636, t = 3.066). Moreover, both low and high levels of professional commitment were tested, and the slope difference was also significant regarding procedural justice (t = 4.67). Present study findings were also consistent with the findings of [127] professional commitment mostly adverse dealing with the behavior of knowledge hiding. Thus, H5a was supported.

Afterwards, we test the distributive justice and professional commitment direct effect on knowledge hiding. Results revealed that distributive justice direct effect on knowledge hiding was statistically substantial (F = 12.887), and the interaction effect between distributive justice and professional commitment (distributive justice x professional commitment) on knowledge hiding are also statistically substantial (β = 0.109, t = 2.029). Via using dummy variables, the moderator variable dataset was divided into two groups; low and high. So, we test the distributive justice on knowledge hiding at both low and high levels of professional commitment. As expected, distributive justice negatively affects knowledge hiding while having a high-level professional commitment (β = −0.256, t = −4.118). In comparison, distributive justice is positively related to knowledge hiding while having low-level professional commitment (β = 0.216, t = 2.203). Moreover, low and high levels of professional commitment were tested, and the slope difference was also significant regarding distributive justice (t = 3.66). Present study findings were also consistent with the findings of [127] because professional commitment mostly adverse dealing with the behavior of knowledge hiding. Thus, H5b was supported.

**Fig. 3:**
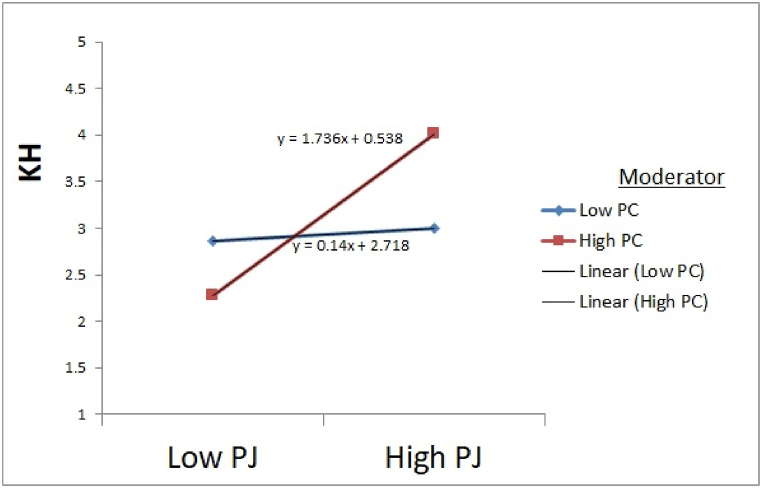
Moderation effects of Professional Commitment on Knowledge Hiding through Procedural Justice. Source: Based on Game's Gaskin Statistical Package.

**Fig. 4:**
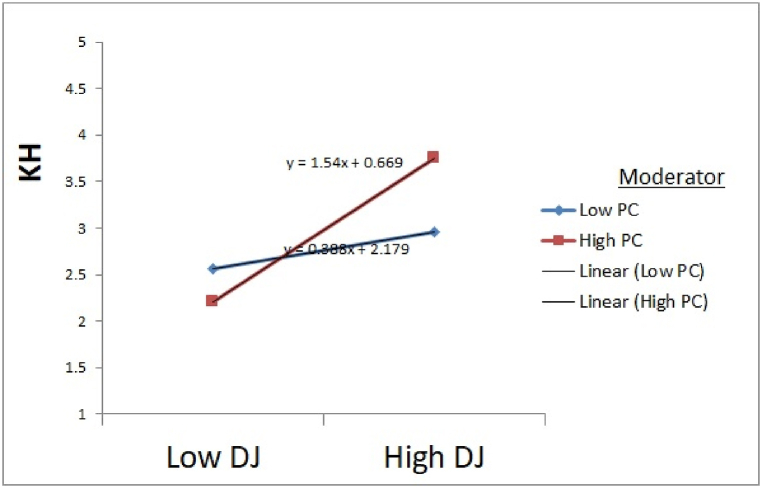
Moderation effects of Professional Commitment on Knowledge Hiding through Distributive Justice. Source: Based on Game's Gaskin Statistical Package.

**Fig. 5:**
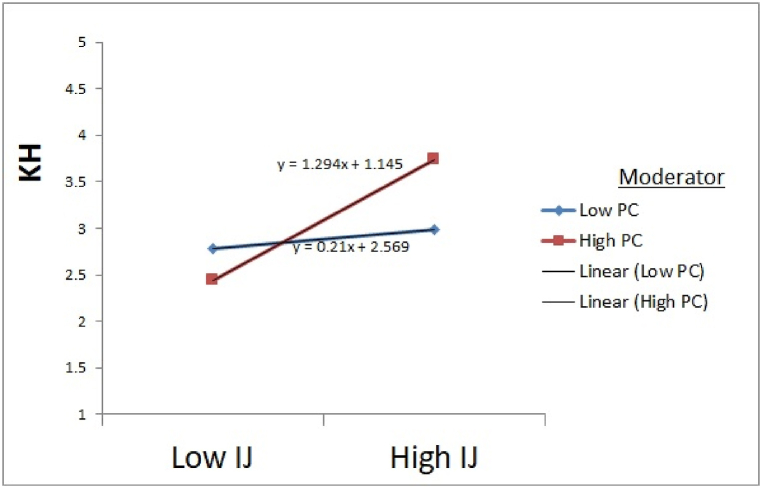
Moderation effects of Professional Commitment on Knowledge Hiding through Interactive Justice. Source: Based on Game's Gaskin Statistical Package.

Finally, we test the interactional justice and professional commitment direct effect on knowledge hiding. Results revealed that interactional justice direct effect on knowledge hiding was statistically substantial (F = 9.867), and interaction effect among interactional justice and professional commitment (interactional justice x professional commitment) on knowledge hiding are also statistically substantial (β = 0.101, t = 2.009). Via using dummy variables, the moderator variable dataset was divided into two groups; low and high. So, we test the interactional justice on knowledge hiding at both low and high levels of professional commitment. Interactional justice, as expected, negatively affects knowledge hiding while having a high-level professional commitment (β = −0.544, t = −5.807). In contrast, interactional justice is positively related to knowledge hiding while having low-level professional commitment (β = 0.512, t = 2.491). Moreover, both low and high levels of professional commitment were tested, and the slope difference was also significant regarding interactional justice (t = 5.51) as shown in Fig. 3, Fig. 4, Fig. 5 respectively. Present study findings were also consistent with the findings of [127] because professional commitment mostly adverse dealing with the behavior of knowledge hiding. Thus, H5c was also supported.

## 5 Discussions on findings

The present study suggests numerous theoretical intuitions towards job performance. Current research empirically investigated organizational justice facets and their dynamic appliance in an organization. Finding the association among organizational justice facets and in-role and innovative performance through both mediations of knowledge hiding and well-being and professional commitment as a moderator has valuable implications in both perspective of theoretical and as well as practical. Initially, results revealed that procedural, distributive and interactional justice negatively impact knowledge hiding, affirming the findings of extant studies in the organizational domain. The results of present research are accordant with the findings of [128,129] where they institute that organizational justice positively adaptation the behavior of knowledge hiding that ultimately trigger the positive behavior toward innovative performance. Present work provides a detrimental impact of knowledge hiding on employees' well-being as well as on the organization [130] are also consistent with the findings of recent study [131]. A contradictory relation exists between knowledge hiding and well-being, and also with job performance because knowledge hiding negatively impacts employees' well-being.

Whereas, well-being positively affects job performance. Results revealed that professional commitment contributed positively to the relationship between organizational justice facets and knowledge hiding [43]. A similar phenomenon exists among other domains (e.g., professional individuals, students) regarding the psychological factor of knowledge hiding and knowledge sharing. Thus, knowledge hiding facets— playing dumb, evasive hiding, and the rationalized hiding existed among potential employees was also proved in the findings of recent study on knowledge hiding and psychological strain [132]. Still, the justice-related phenomenon may vary in the present study, as highlighted in extant studies.

Findings of the study suggest that if an employee alleged procedural, distributive and interactional justice, their involvement in knowledge hiding might be minor rendering to answer the first hypothesis. Consequently, findings of the current study were quite familiar with the results of [133] which also revealed that justice greatly hinder the mechanism of knowledge hiding. The core competence of the organization is the employee's knowledge, and its exchange is beneficial for both organization and employees as well so, organizations are provoked with much budgets due to knowledge management difficulties [128]. According to Ref. [134], senior employees played an exemplary role for potential employees, so they adopted the senior employees' behavior to treat others. To gain trust and a comfortable environment among employees and organization, organizational justice facets played a remedial role. Potential employees shift their intention toward knowledge sharing than knowledge hiding [8]. Additionally, once the seniors established organizational justice and degraded injustice, employees discontinued knowledge hiding behavior. Notably, findings indicated that in-between the negative association of organizational justice facets and knowledge hiding, professional commitment plays a moderator role that weaken the direct relationship when professional commitment is high. Present study results are also aligned with the extant study findings that professional commitment played a vital role on the relationship among organizational justice and knowledge hiding [86]. Therefore, knowledge hiding among potential employees is redundant in the justice environment because it effortlessly exposes knowledge hiding.

Based on the present study results, knowledge hiding negatively mediates employees' well-being, responding to the second hypothesis. Employees who have the behavior of knowledge hiding can create hurdles in other employees' well-being. As for hypotheses, three (a) and three (b), well-being positively affecting employees' in-role and innovative performance was also justified on behalf of present findings. Previous research engrossed on the antecedents and consequences of innovative behavior of employees [32] but overlooked the practice of knowledge hiding that paved a way for well-being so, present research explores it through the facets of organizational justice. Finally, the moderating hypothesis revealed that professional commitment is significantly fortified in the context of interactional justice while comparing the other two domains and recommends that employees with high professional commitment have a lower perception of harm via knowledge hiding. Present study findings are consistent with the extant studies that professionally committed employees purely focus on the norms and ignore immoral practice because professional commitment leads toward social responsibility [135]. Instinctively, professional commitment changes the psyche of seniors regarding career development, and they frankly deal with their work fellows and subordinates as a companion rather than entrants. So, highly professional committed employees were concerned with a supportive climate and ignored the knowledge hiding through justice; contrarily, lower professionally committed employees might interact with living approach and deals with work fellows as entrants through incorporating injustice highlighted in Fig. 2.

### 5.1 Theoretical contribution

Based on the signifying factors and knowledge hiding dynamic mechanism between employees, theoretical contributions are evoked. The present study instructs the trustworthy admittance to preclude the behavior of knowledge hiding among employees markedly. This study used psychological ownership knowledge theory by Ref. [29] and social exchange theory by Ref. [136] to support the theoretical framework. So, examining the mediating role of knowledge hiding contribute to the body of literature with support of social exchange and psychological ownership knowledge theories.

Present research offers insightful outcomes in an organizational context that gained little attention on employees' knowledge hiding. Empirical evidence revealed the behavioral impact of knowledge hiding among employees; however, present research deals it with the facets of organizational justice and professional commitment that ultimately paved the robust way of optimistic performance that has been comparatively limited. Initially, we contributed to the literature by enlightening professional commitment as a moderator on the association between organizational justice and knowledge hiding. While considering the extant study, researcher take organizational commitment as an influencer toward knowledge hiding in organizational perspective [86]. It can advance the latitude of organizational justice facets via demonstrating honesty, trust, sacrifices, and unselfishness through their mechanism that developed their work fellow's relational behavior. Such contribution via moderating professional commitment on organizational justice facets and knowledge hiding is the need of the hour at present stage.

Secondly, this study contributes to the available literature by measuring the mediational role that suggests the positive influence of well-being toward employees in-role and innovative performance and the negative effect of knowledge hiding between organizational justice facets and employee's well-being. In the shadow of social exchange theory, extant study findings also revealed that an optimistic association exist among well-being and the employee innovative behavior [32]. Nevertheless, knowledge hiding plays a negative mediating in-between organizational justice facets and well-being that bridge the gap prevails in the available literature of knowledge hiding. Still, rare studies exist in the past that examine well-being as a mediator between knowledge hiding and job performance facets [43]. Presently, we bridge the scientific linkages of knowledge hiding consequences and offer well-being as a budding means for encouraging in-role and innovative performance.

Thirdly, research was scarce on the association between organizational justice facets and job performance through knowledge hiding. Explicitly, very little evidence prevailed about the organizational justice facets direct association with knowledge hiding and indirect linkage with in-role and innovative performance. Thus, the study contributed to the body of knowledge by linking knowledge hiding with life, workplace and psychological well-being [18] by hypothesizing professional commitment, as a moderating factor, among the association of organizational justice facets and knowledge hiding.

Finally, the outcomes of this research showed that distributive justice had greater adverse effects on the knowledge hiding in comparison to procedural and interactional justice, so we focus on perception toward organizational justice in a behavioral context. Similarly, this study provides a shred of evidence that professional commitment highly moderates distributive justice as compared to others and remarkably influences the indirect relationship among organizational justice facets and knowledge hiding. Also, this research suggests future studies to examine the interactional justice link with knowledge hiding via professional commitment of employees [85,86]. Hence, this study is imperative because it helps future studies to examine the influence of commitment to improve employee's perception about organizational justice to freeze knowledge hiding.

### 5.2 Implications for employees and organizations

Based on multiethnic employees in organizations, the present study suggests numerous insights toward knowledge hiding behavior. First, this research highlighted valued implications that how managers and senior employees undermine the concept of knowledge hiding among employees through incorporating justice. The managers and senior employees can do so by indicating procedural, distributive and interactional justice through their activities to stimulate their subordinates to emulate their actions that ultimately building a cooperative relationship among employees. Consequently, higher management and decision-makers of an organization need to be stringent on practicing the justice mechanism for employee's well-being particularly, in assessment and remuneration procedure that eventually stimulate the employee performance. Such cooperative relationships between employees would encourage an altruistic approach for their associate and initiate the tradition of knowledge sharing instead of knowledge hiding. Practical implications of present study were also consistent with extant studies that employees' behavior of knowledge hiding sanctuary their vitalness [137] and narrow-minded intentions urge the knowledge hiders to hide the knowledge from peers [138], however, knowledge transferers played a remedial role for their peers that ultimately beneficial for the organizations [139].

Second, the managers and senior employees need to consider the knowledge hiding facets— playing dumb, evasive hiding and rationalized hiding while mending their behavior. A customized concern and poor management refer to the diverse configuration of knowledge hiding. It became the need of the hour to identify the start-up behavior of knowledge hiding. Third, regarding managerial point-of-view, they must emphasize adopting different employee practices to improve the direct effect of organizational justice on employees. Generally, organization administrators' dire need to comprehend the significance of organizational facets to advance the employees performance and more specifically, organizational administrations practice of organizational justice must endeavor to advance a deliberate and organized strategy in the workplace [140]. Therefore, managers have to identify employees with the higher level of professional commitment and a good relationship with their subordinates to handle the knowledge hiding smoothly. Based on the professional commitment, different reactions are indicated by the employee's perception toward managers and senior employees, so different implication levels of justice discourage knowledge hiding behavior.

Forth, where there is a concern of resources allocation, there must be a shortage of resources in different organizations in diverse circumstances. Considering the perspective of organizational justice facets— procedural, distributive and interactional justice, managers have to practice the unbiased and moral grounds to sustain the balanced allocation of resources. According to Ref. [141], such practice of management triggers the organizational citizenship behavior among employees that ultimately enhance the insights of organizational justice that positively contribute toward employee performance. Therefore, adoption of modern and individual focused techniques helpful for reshaping the employee's perception towards organizational justice.

Management and owners of the organization must recognize their responsibilities and take it seriously under the shadow of knowledge hiding. The perfect demotion practice of knowledge hiding behaviors if prevailed in an organization than ultimately the kind practice of knowledge sharing will flourish and eventually be the cause of employees' performance enhancement. Organizational justice put valuable contribution among performance related conditions and both intervening constructs of knowledge hiding and employees’ well-being played a game changing role that proved to be beneficial for organization and as well as employees. Consequently, organizational management must consider the attitude of ecofriendly for the sake of acknowledging the practice of knowledge sharing and demolishing the behavior of knowledge hiding.

Finally, managers have to give more intention towards improvement of employee's professional commitment to reduce the knowledge hiding. Because, commitment enable their compassionate beliefs, altruism and honesty towards their professional responsibilities. So, during recruitment process of employee's capability asses their approach towards their job responsibilities must be observed. Present study recommends that recruitment away from the contemplation for employees' capability to comprehend their professional commitment. In the organizational context, personal background, organizational commitment and support, and degree of autonomy overwork are the critical elements of professional commitment [142]. We also suggested that via assimilating knowledge sharing into organizational justice, that might be assisted to frequently clasp the knowledge sharing that ultimately encourage the mysterious instead of already known knowledge.

### 5.3 Limitations and future research

There are numerous contributions and implications in present research toward literature, and there should be some limitations. Knowledge hiding is environment-based phenomena, since in some professional and technology-based organization hiding is important to accelerate and manage the sustainability and overall performance. First, study findings are based on the same source data collection with the time-lagged survey. According to Ref. [97], data collected through single instrument may have chances to be affected by common method biases. So, we suggest implementing a longitudinal study for future research for more clarification of casual relationship. Second, data were collected only from Malaysian organizations that can make limitized the findings' generalizability so, it could not reflect the clear picture of employee's perception about organizational justice. Because a considerable variation exists among Malaysian, other developing countries and Western settings that ultimately influence the employees' mentality. Third, this research is based on the self-reporting mechanism of knowledge hiding as suggested by existing studies [13,143] and while the assessment of the performance of employees, practitioners and potential researchers still try to discover the complications in references that can undoubtedly define the performance as a productivity or process [144]. Organizational justice and the performance of employees' research is a significant feature of reviewing the science of organizational behavior and while measuring the employee performance, it weighed over self-evaluated the attainment of work aptitudes in booming the work roles and knowledge while doing perform their duties. Fourth, present research major limitation is that it based on the random sampling techniques because taking sample from population under this sampling technique is time consuming and cost deficient. Fifth, this study takes two months lag time to conduct data collection and used dual wave survey that also be a problematic and hectic procedure, more conveniently, cross-sectional data will be an easy approach for data gathering in social sciences research. Finally, present research considerable limitation is the use of structural equation modelling, because mostly researcher prefer to use PLS-SEM instead of structural equation modelling and as well as no control variable was used because such type of variable mostly change the scenario and paved a way for better generalizability. So, this study suggests valued insights toward the existing relationships of knowledge hiding among employees.

Additionally, in the perspective of organizational justice, study focused on managers and senior employees. In future, data will also collect from higher management to commencing the knowledge hiding effects on various layers of management. A meta-analysis can be painstaking to upsurge the generalizability of the research and by taking other countries like Pakistan, and India, comparative study will be conducted for generalization. Finally, organizational justices’ facets correspond with professional commitment will demoralize the biting behavior of knowledge hiding. For future perspective, take into account supplementary constructs like benevolence, empowering leadership, and trust will also be the cause of change in employee performance. Additionally, well-being put prospective mediating tools among knowledge hiding and employees' in-role and innovative performance that offer an avenue for further research.

### 5.4 Conclusion

The phenomenon of KH prevailed in all over the world and specifically in Asian context. Manufacturing industries in Malaysia need to eliminate this behavior and promote the concept of knowledge sharing. For that purpose, organizational justice facets, professional commitment, and the role of expert employees played a remedial role for employee's well-being that paved a way for success of organization and as well as workforce.

## Ethics statement

No animal studies are presented in this manuscript. “This study involving human participants was reviewed and approved by the Ethics Committee of the Department of Management Sciences, Universiti Sultan Zainal Abidin (UniSZA), Terengganu, Malaysia. The patients/participants provided their written informed consent to participate in this study.”

## Author contribution statement

1) conceived and designed the experiments; Asad Ur Rehman and Irfan Sabir
2) analyzed and interpreted the data; Asyraf Afthanorhan
3) contributed reagents, materials, analysis tools or data; Abdul Rauf
4) wrote the paper: Hamid Mahmood

## Data availability statement

Data will be made available on request.

## Declaration of competing interest

The authors declare that they have no known competing financial interests or personal relationships that could have appeared to influence the work reported in this paper.
